# Draft Genome Sequences of Two Benthic Cyanobacteria, *Oscillatoriales* USR 001 and *Nostoc* sp. MBR 210, Isolated from Tropical Freshwater Lakes

**DOI:** 10.1128/genomeA.01115-16

**Published:** 2016-10-13

**Authors:** Shu Harn Te, Boon Fei Tan, Janelle R. Thompson, Karina Yew-Hoong Gin

**Affiliations:** a NUS Environmental Research Institute, National University of Singapore, Singapore, Singapore; b Centre for Environmental Sensing and Modelling, Singapore-MIT Alliance for Research and Technology, Singapore, Singapore; c Department of Civil and Environmental Engineering, National University of Singapore, Singapore, Singapore

## Abstract

Genomes of two filamentous benthic cyanobacteria were obtained from cocultures obtained from two freshwater lakes. The cultures were obtained by first growing cyanobacterial trichome on solid medium, followed by subculturing in freshwater media. Subsequent shotgun sequencing, *de novo* assembly, and genomic binning yielded almost complete genomes of *Oscillatoriales* USR 001 and *Nostoc* sp. MBR 210.

## GENOME ANNOUNCEMENT

Benthic cyanobacteria inhabit the bottom of a diverse range of bodies of water, including lakes, wetlands, estuaries, and oceans, forming benthic mats in these environments (1). Under favorable conditions, they can proliferate rapidly and synthesize undesirable secondary metabolites, including toxins and odors (2). However, they are less studied compared to planktonic cyanobacteria even though they are able to cause similar ecological and water quality impacts on affected waters (3). Two filamentous benthic cyanobacteria isolated from tropical freshwater lakes in Singapore were identified as *Oscillatoriales* USR 001 and *Nostoc* sp. MBR 210, based on morphological traits (4). Here, we present additional genomic information about these isolates, which is important for functional annotation, pathways analysis, comparative genomics and for better understanding of their roles in bloom formation.

The two filamentous cyanobacteria were acquired through an agar culturing method (5). Briefly, lake sediment samples were streaked across agar plates enriched with McBride *Listeria* agar (MLA) medium (6). The agar plates were incubated (25°C, light intensity: 25 µmol/m^2^s) until green filaments appeared; then individual filaments were aseptically cut, transferred, and cultivated in sterile MLA media for 2 weeks. The genomic DNA extraction, Illumina HiSeq 2000 sequencing, and read quality controls were conducted following a method described previously (7). Subsequently, the two metagenomes were *de novo* assembled separately into scaffolds using CLC Genomics Workbench version 8. Contigs belonging to *Cyanobacteria* in each metagenome were separated from those of heterotrophic bacteria using MetaBAT (8), following which genome completeness and sequence contaminants were determined using CheckM (9), and the lack of a sequence contaminant was confirmed using a BLAST-based approach (10). The two genomes were annotated using RAST (11) and NCBI PGAP (http://www.ncbi.nlm.nih.gov/genome/annotation_prok).

Genomes of the two cyanobacteria have GC contents of 41% and completeness of 99%, assessed using checkM by comparing 579 to 583 reference marker genes in 79 to 82 lineage-specific reference genomes. The draft genome for *Oscillatoriales* USR 001 comprises 5.9 Mbp contained in 96 scaffolds, while *Nostoc* sp. MBR 210 has a genome size of 6.9 Mbp contained in 36 scaffolds. The 16S rRNA of *Nostoc* sp. MBR 210 (1,482 bp) is 99% identical to that of *Nostoc piscinale* CENA21 (CP012036.1), whereas the 16S rRNA of *Oscillatoriales* USR 001 (1,492 bp) is 98% identical to three members of the family *Oscillatoriales*: *Kamptonema animale* (EF654087.1), *Phormidium animale* CCAP (HF678514.1), and *Oscillatoria lutea* (KM019965.1). Further comparison between the genome of *Oscillatoriales* USR 001 with all reference genomes of the three genera currently available in the NCBI and JGI IMG databases (12) revealed a two-way average nucleotide identity of <90%, precluding the classification of USR 001 to the genus level. As members of these two organisms are known to be potential toxin (microcystin and anatoxin) and odor (geosmin and 2-methylisoborneol) producers, we used antiSMASH version 3.0.5 (13) and BLASTp to search for potential gene- or gene cluster–encoding secondary metabolites, to which no toxin and off-flavor-producing gene (as above) was identified in both genomes. Both genomes carry genes for photosynthesis and CO_2_ and nitrogen fixation (e.g., carboxysome and nitrogenase, respectively) and a limited number of genes encoding sugar utilization (e.g., beta-galactosidase in USR 001; alpha-mannosidase for mannose utilization in MBR 210), suggesting their potential roles as photoheterotrophic nitrogen fixers.

### Accession number(s).

These whole-genome shotgun projects have been deposited in DDBJ/ENA/GenBank under the accession numbers MBRE00000000 (*Oscillatoriales* USR 001) and MBRD00000000 (*Nostoc* sp. MBR 210). The versions described in this paper are the first versions, MBRE01000000 and MBRD01000000.
